# Distribution Characteristics and Influencing Factors of Organochlorine Pesticides in Agricultural Soil from Xiamen City

**DOI:** 10.3390/ijerph20031916

**Published:** 2023-01-20

**Authors:** Ziqiang Gao, Yixian Chen, Qijin Qin, Rui Wang, Zhineng Dai

**Affiliations:** 1 School of Environmental Science and Technology, Xiamen University of Technology, Xiamen 361024, China; 2 Key Laboratory of Environmental Biotechnology (XMUT), Fujian Province University, Xiamen 361024, China

**Keywords:** organochlorine pesticides, soil, distribution characteristics

## Abstract

The concentration and distribution of 15 organochlorine pesticides in the soil of Xiamen City were determined. Overall, among the 15 selected target pesticides, 14 organochlorine pesticides (OCPs) were detected (hexachlorobenzene was not). The range of detected pesticides was undetected−10.04 ng/g, the total detection rate was 35.2%, and the three pollutants with the highest detection rate in all samples were Heptachlor (66.7%), δ-Hexachlorocyclohexane (60.0%), and p, p′-Dichlorodiphenyldichloroethane (58.3%). The degree of pollution in descending order was Tong’an > Xiang’an > Jimei > Haicang. Linear regression analysis of soil properties and OCP concentration distribution revealed that OCPs were positively correlated with water content, dissolved organic carbon, and pH and negatively correlated with cation exchange capacity. The origin of OCPs was evaluated using the relationship between the parent compound and its metabolites, with possible new hexachlorocyclohexane and isomer (HCHs) input. By comparing the risk screening values of ΣHCHs and ΣDDTs in “Soil Environmental Quality Standards”, the concentrations in all soil samples were lower than the standard values, which indicated that the OCP residues in Xiamen were very low.

## 1. Introduction

Since the 1950s, OCPs have been widely produced and consumed in large quantities due to their cheapness and high efficiency, mainly for agricultural use and vector control [1,2]. The use of HCH technology in agriculture was banned until the 1980s, followed by Dichlorodiphenyldichloroethane and its isomers (DDTs) and Lindane in the 1990s [1]. In the target insects, OCPs act on sodium channels on the axons of neurons and thereby disrupt the transmission of “action potentials” across the nerves; it causes uncontrolled, repetitive, and spontaneous firing along the axons of neurons, which often causes the muscles used for breathing to lose control and the insect to suffocate [3]. For humans, the EPA lists the following human health hazards from DDT and its derivatives: damage to the liver; nerve damage: temporary nervous system damage; reproductive system damage [3,4,5]. The continuous exposure of OCPs to human ovarian surface epithelial cells will secrete various pro-inflammatory cytokines and form a microenvironment that promotes tumor, further enhancing genetic instability (DNA damage). These results illustrate the carcinogenic potential of OCPs in the transformation of epithelial ovarian cells [6]. Although the 2001 Stockholm Convention on Persistent Organic Pollutants (POPs) included nine OCPs in the so-called “dirty dozen POPs”, including aldrin, dieldrin, heptachlor, etc., OCPs are still detected in large numbers in the environment [7].

Even though these compounds were phased out decades ago, based on the C-Cl (carbon or fluorine or bromine bond) covalent bond they have, they are very stable in the environment and lack biodegradability [3]. After application, pesticides may be washed from plants and crops to the soil surface by rainwater, and runoff is a major source of river pollution. Direct volatilization of organic compounds from surface water, soil, and plants is also possible [8]. Once pesticides enter the soil, they are either “absorbed” by the soil and thus temporarily unavailable, or they are moved and transported with soil particles through erosion processes, which include reduction induced by soil microbes or conversion to other simpler molecules [9,10]. It has been pointed out in the literature that OCPs easily combine with organic matter and move with it [11]. However, the influence of other soil properties on OCPs is still unknown, so it is necessary to study the influence of different soil properties on the distribution of OCPs.

Some studies have evaluated the harmful effects of organochlorine pesticide pollution on human health; hazard ratios on the basis of 95th percentile concentrations were exceeded for most of the investigated OCPs in rice and wheat, which indicated that the consumption of contaminated cereal crops poses a serious risk to the health of the population in the study area [12]. Xiamen is a special economic zone in China and an important central city on the southeast coast. It is located in East China and has a permanent population of 5.28 million. There have been studies on the determination of PCBs and OCPs in shellfish in Xiamen; the previous results showed that DDT was the main OCP, followed by aldrinoids and endosulfan. Although the concentration of DDTs is increasing year by year, the content of aldrin and endosulfan is increasing and has a higher risk of carcinogenicity [13]. Soil is also the carrier of land crops, and there is no research on the content of organochlorine pesticides in Xiamen. In order to better understand the distribution of OCPs in the environment of Xiamen City and the influence of soil properties on it, we sampled soils from different plots. The aim of this study was to investigate the residues of OCPs in agricultural soils in Xiamen City and examine the influence of relevant soil properties on their distribution, which may help protect the public from potential health hazards.

## 2. Materials and Methods

### 2.1. Sample Collection and Extraction

Ten crop planting bases in each block of Xiamen City were selected as the sampling points; the geographical locations are given in Supplementary Table S1. The soil samples at each sampling point were composed of 3 sub-samples, which were from 0–10, 10–20, and 20–30 cm soil layers. These samples were taken from 20 × 20 cm^2^. All samples were collected from November 2021 to January 2022. After removing large rocks and plant roots, they were put into polyethylene plastic bags, transported to the laboratory on that day, and stored in a 4 °C refrigerator after unified numbering.

The sample was extracted according to the methods provided by Tor et al. [14], Vagi et al. [15], and Aiyesanmi et al. [16] and improved according to the methods in the national standard (Soil and Sediment Extraction of Organic Compounds Ultrasonic Extraction HJ 911–2017). After thawing and air drying, foreign matters such as plant branches, leaves, and stones were removed on a stainless steel plate, and the soil samples were mixed. After adding a certain amount of anhydrous sodium sulfate (depending on the moisture content of the sample; the total amount should not exceed half of the beaker) to the soil sample (about 20 g), it was stirred evenly with a horn spoon. Then, about 50 mL of dichloromethane was added into the beaker containing the sample, resulting in the liquid level of the extractant being about 2 cm higher than the surface of the solid sample, and the sample was ultrasonically extracted for 30 min. The above operations were repeated twice to extract the soil samples, and the three extracts were combined. Finally, the combined extracts were placed in a rotary evaporator, evaporated to nearly dryness in a water bath at 40 °C, and then diluted to 1 mL with n-hexane.

### 2.2. Instrumental Analysis

The quantitative determination of organochlorine pesticides was performed by a Shimadzu GC-2010 gas chromatograph equipped with an HP-5 capillary column. The instrument parameters were set according to the methods of Zhao et al. [17] and Can-Güven et al. [18] and were optimized based on preliminary measurements. The GC system was operated in a splitless mode. Each sample injection volume was 1.0 μL. Injection temperature was maintained at 260 °C. Nitrogen was used as the carrier gas at 2.0 mL/min in the constant flow mode. The temperature started at 100 °C, then increased to 220 °C/min at a rate of 30 °C/min, followed by a ramp up to 240 °C/min at a rate of 4 °C/min, then to 260 °C/min at a rate of 10 °C/min (held for 10 min). The tail gas blowing flow rate was kept at 30 mL/min.

### 2.3. Quality Control and Quality Assurance

The mixed standard samples of organochlorine pesticides were purchased from China Institute of Metrology. The standard substance used high-purity isooctane/high-purity toluene (mass ratio: 99.7/0.3) as the solvent, and α-Hexachlorocyclohexane (α-HCH), δ-Hexachlorocyclohexane (δ-HCH), γ-Hexachlorocyclohexane (γ-HCH), δ-Hexachlorocyclohexane (δ-HCH), o, p′-Dichlorodiphenyltrichloroethane (o, p′-DDT), p, p′-Dichlorodiphenyltrichloroethane (p, p′-DDT), p, p′- Dichlorodiphenyldichloroethane (p, p′-DDD), p, p′-Dichlorodiphenylethane (p, p′-DDE), hexachlorobenzene, heptachlor, heptachlor epoxide, aldrin, dieldrin, endrin, and mirex, a total of 15 organochlorine pesticides. The standard solution was prepared into 0, 5, 10, 20, and 50 μg/L standard solutions with n-hexane, and a standard curve was established. The correlation coefficients of the established standard curves were all between 0.938–0.999, which meets the requirements. The linear regression equation and fitting degree are given in the Supplementary Table S2.

### 2.4. Statistical Analysis

The content of OCPs in soil was characterized by the descriptive statistical method, and all statistical analysis was performed using IBM SPSS Statistics 22 (International Business Machines Corporation, Armonk, NY, USA). The reported values include the range, mean, and standard deviation of OCPs in each soil sample. Histograms of organochlorine pesticide concentrations and linear regression plots with soil property parameters are described by Origin 2018 (OriginLab Corporation, Northampton, MA, USA).

## 3. Results and Discussion

### 3.1. General Situation Regarding Agricultural Soil (Distribution Characteristics of OCPs in Agricultural Soils of Xiamen)

The descriptive statistics of the detection of organochlorine pesticides in soil samples are shown in Table 1. In this investigation, 14 kinds of organochlorine pesticides were detected out of the 15 target pesticides selected, with the exception being hexachlorobenzene. The range of detected pesticides was n.d.–10.04 ng/g, the average value was 0.149 ng/g, and the total detection rate was 35.2%. Among them, the highest pollutant concentration detected in a single sample was 10.04 ng/g, and the three pollutants with the highest detection rate in all samples were Heptachlor (66.7%), δ-HCH (60%), and p, p′-DDD (58.3%).

There are eight isomers of HCHs; the most common four are α-, β-, γ-, and δ-HCHs. The most common of these is g-HCH (also known as lindane), which is usually the major isomer [19]. The concentrations of ΣHCHs (sum of α-HCH, β-HCH, γ-HCH, and δ-HCH) varied from n.d. to 0.78 ng/g, with an average value of 0.076 ng/g. Among these compounds, δ-HCH was generally the most dominant, with concentrations ranging from n.d. to 0.78 ng/L, with a mean value of 0.223 ng/g at a 60% detection frequency of the soil samples. Compared with other regions, the pollution levels of HCHs in agricultural soils in Xiamen were lower than those in northern regions (34.0 ng/g) [20], central regions (15.39 ng/g) [21], Shanghai (2.41 ng/g) [22], Zhangzhou (9.79 ng/g) [23], Qinghai-Tibet Plateau (0.35 ng/g) [24], and other countries such as South Korea (0.2 ng/g) [25]; they were higher than in Kuwait (0.065 ng/g) [26] and Mexico (0.027 ng/g) [27].

Dichlorodiphenyltrichloroethane (DDT) is converted into Dichlorodiphenyldichloroethane (DDD) and Dichlorodiphenylethane (DDE), respectively under anaerobic and aerobic conditions, and their derivatives include o, p′-DDT, which is made from a racemic mixture of enantiomers (1:1) [28]. The concentrations of ΣDDTs (sum of o, p′-DDT, p, p′-DDT, p, p′-DDD, and p, p′-DDE) varied from n.d. to 10.04 ng/g, with an average value of 0.232 ng/g. As the main product of DDT after dechlorination, DDD was found at a higher concentration than the parent and was detected at a higher frequency in soil samples. The pollutant level of DDTs is lower than that in the north (30.0 ng/g) [20] and central (151.56 ng/g) [21] areas, Shanghai (21.41 ng/g) [22], Zhangzhou (3.86 ng/g) [23], Qinghai Tibet Plateau (1.36 ng/g) [24], and other countries such as South Korea (23.18 ng/g; [25], Kuwait (1.22 ng/g) [26], and Mexico (1.6 ng/g) [27].

Other organochlorines are grouped together, including cyclodiene organochlorines and mirex. The concentrations of ΣOOCPs (sum of hexachlorobenzene, heptachlor, heptachlor epoxide, mirex, aldrin, dieldrin, endrin) varied from n.d. to 3.74 ng/g, with a mean value of 0.144 ng/g. For cyclodiene organochlorine pesticides, except epoxyheptachlor as a metabolite, the average concentrations of heptachlor, aldrin, dieldrin, and endrin were all higher than 0.15 ng/g, and the detection rates were higher than 45%, which may be due to the strong adsorption of cyclodiene to soil and sediment, resisting leaching to groundwater and persisting for a long period of time.

**Figure 1:**
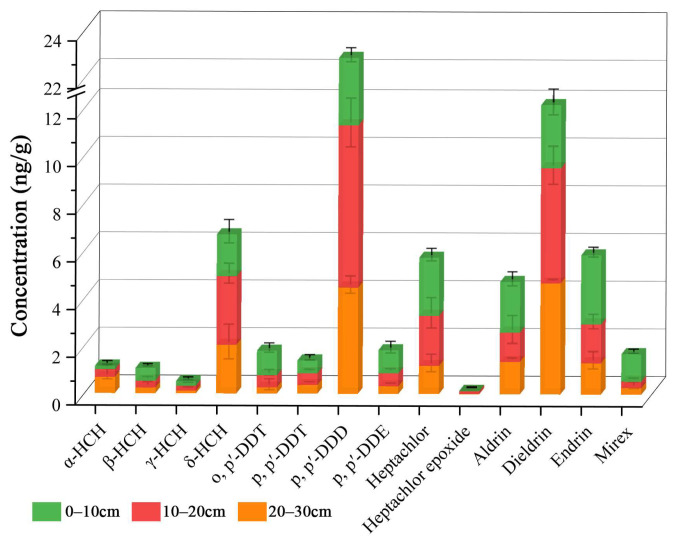
Vertical distribution concentration of 14 organochlorine pesticides.

The vertical distribution concentration range (0–30 cm) of HCHs in soil are respectively n.d.–0.735 ng/g (mean = 0.264 ng/g), 0.01–0.8 ng/g (0.368 ng/g), and 0.025–0.65 ng/g (0.308 ng/g) (Figure 1). HCHs were detected only at three sampling points on the soil surface, and at least one HCH was detected after the depth increased. It seems that HCHs do not easily stay on the soil surface; a large number of HCHs are detected in the middle layer, and the concentration is high. The vertical distribution concentration range of DDTs in soil is 0.08–10.04 ng/g (1.412 ng/g), n.d.–5.27 ng/g (0.834 ng/g), and n.d.–3.815 ng/g (0.540 ng/g), respectively. DDTs were detected in the surface layer of all soil samples, and the highest concentration of all target pollutants was detected. With the increase of depth, the average concentration and detection rate of DDTs decreased. The vertical concentration ranges of OOCPs in soil were n.d.–3.515 ng/g (1.131 ng/g), 0.185–3.22 ng/g (1.018 ng/g), and n.d.–4.07 ng/g (0.88 ng/g). The distribution of OOCPs is similar to that of DDTs. There is a higher concentration detected on the soil surface, and the detected concentration decreases with the increase of depth.

**Figure 2:**
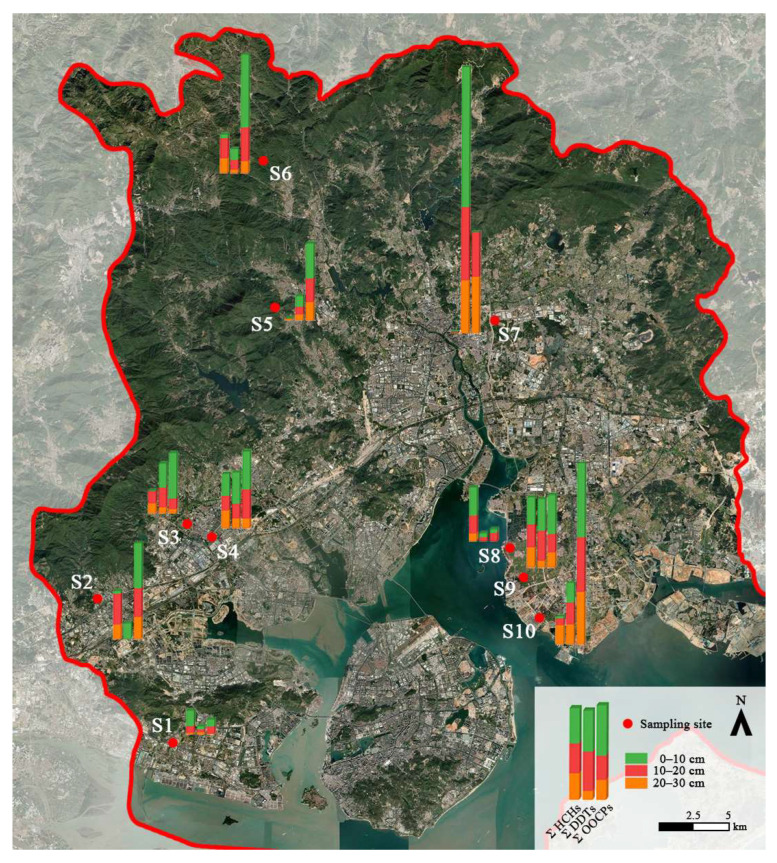
The spatial distribution of OCPs in agricultural soil of Xiamen.

In order to meet the horizontal coverage detection of agricultural soil in Xiamen, the sampling point involves four administrative regions (except Huli District and Siming District; two blocks that have almost no agricultural land), including Haicang District (S1, S2), Jimei District (S3, S4), Tong’an District (S5, S6, S7) and Xiang’an District (S8, S9, S10) (Figure 2). From the four regions, the order of mean residual concentration of HCHs is: Xiang’an District (1.388 ng/g) > Jimei District (0.995 ng/g) > Haicang District (0.933 ng/g) > Tong’an District (0.458 ng/g). The order of mean residual concentration of DDTs is: Tong’an District (6.832 ng/g) > Jimei District (1.368 ng/g) > Xiang’an District (1.338 ng/g) > Haicang District (0.308 ng/g). The order of mean value of residual concentration of OOCPs is: Tong’an District (4.343 ng/g) > Xiang’an District (3.603 ng/g) > Jimei District (1.780 ng/g) > Haicang District (1.440 ng/g). Overall, the degree of pollution in descending order is Tong’an District > Xiang’an District > Jimei District > Haicang District, among which Tong’an District and Xiang’an District are more seriously polluted than the other two districts. From the perspective of Xiamen’s agricultural development, Tong’an District and Xiang’an District account for 86.88% of the irrigated cultivated land area in Xiamen (the main data bulletin of the third national agricultural census in Xiamen City). Obviously, as based on the nature of the soil use, an increase in the frequency of application is also evident. The sampling points with high concentration and high detection rate are mostly concentrated in the northeast of Xiamen City, which may be due to atmospheric transport and deposition. Xiamen has a subtropical monsoon climate, the terrain is high in the west and low in the east, and the wind direction is mostly northeasterly. The atmospheric transport in the southwest is strong, and the drift of the wind will blow pesticides to the non-target area in the form of droplets or dust through the air. In addition, the sampling time is in winter, and radiation fog often appears in the near-ground air layer, causing water vapor condensation, which enhances the dust deposition.

### 3.2. Effects of Soil Properties on Concentration of OCP Pesticides

In this investigation, water content, dissolved organic carbon, pH, and cation exchange capacity were selected as indicators of soil properties, and the characteristics of these factors and the distribution of organochlorine pesticides were studied (Table 2). The moisture ranged from 10.54 to 20.0%, with an average of 15.52%. The pH and dissolved organic carbon (DOC) ranged from 4.34 to 7.27 and 7.25 to 15.19 mg/g, respectively, with the average values of 5.97 and 11.062 mg/g, respectively. The cation exchange capacity ranged from 16.87 to 39.21 cmol/kg, with an average value of 24.107 cmol/kg.

The soil types in Xiamen are mainly red soil and paddy soil. The soil is acidic, and its profile has distinct layers. From top to bottom, it has a humus surface layer, clayey layer, and parent material layer. This kind of soil surface is rich in organic matter, with the increase of depth, the clay content increases, and there is a certain degree of condensation. This may explain why water, pH, and cation exchange capacity increase with depth.

**Figure 3:**
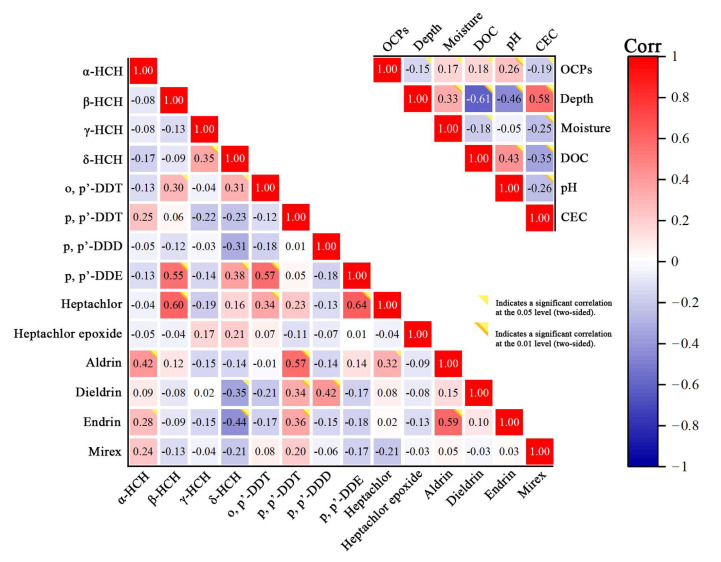
Two-factor correlation analysis between OCPs and soil properties.

In Figure 3, the two-factor correlation analysis of OCPs and sampled soil properties shows that the distribution of OCPs is significantly related to pH (*p* < 0.01) and is also related to water content, dissolved organic carbon, and cation exchange capacity (*p* < 0.05). Soil depth seems to determine the physical and chemical properties of the soil. Because the soil surface is often washed by irrigation and rain, the pH is closer to neutral. At further depths of the soil, there is a decline of the exchange capacity of the soil and air, as well as an increase of the water content in the soil. The increase of clay content leads to the change of the soil structure, which also affects the cation exchange capacity of soil. p, p′-DDT was significantly positively correlated with cyclodiene pesticides (including aldrin, dieldrin and endrin) (*p* < 0.01); these pesticides may come from the same pollution. HCHs were negatively correlated with most pesticides and may have a separate source from other OCPs. The following sections further analyze the impact of various influencing factors on OCPs. The obtained linear regression equation is given in Supplementary Table S3.

**Figure 4:**
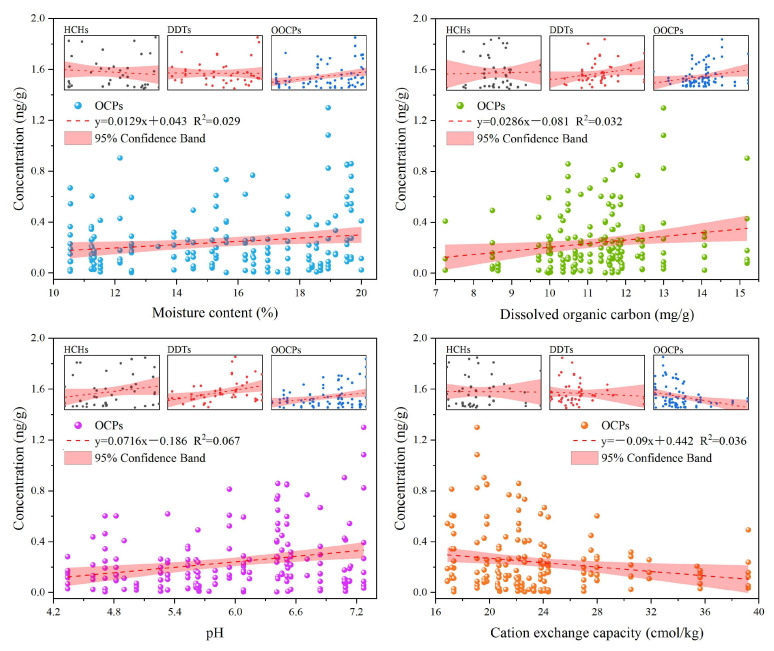
Linear regression analysis between soil properties and OCPs.

According to the 2021 Xiamen City Climate Annual Report [29], the annual precipitation of representative stations in various districts outside Xiamen Island in 2021 are as follows: Tong’an District (1381.3 mm), Haicang District (1062.7 mm), Jimei District (1057.8 mm), Xiang’an District (831.3 mm). Among them, the Tong’an District has the largest proportion of moderate rain and heavy rain, accounting for 16.8% and 10.9%, respectively. Compared with the previous analysis of the pollution degree of each region, precipitation does not seem to be the main factor determining the residue of OCPs. Linear regression analysis of soil properties and OCP concentration distribution (Figure 4) revealed that OCPs were positively correlated with water content, dissolved organic carbon, and pH and negatively correlated with cation exchange capacity (*p* < 0.05). The linear regression equation and goodness of fit are shown in Supplementary Table S3. In the analysis of all influencing factors, the distribution characteristics of HCHs have no characteristic law and have no significant relationship with the slope, being close to 0. It has been pointed out that HCHs tend to migrate downward from the humus layer in the form of free dissolution, while DDTs tend to migrate together with dissolved organic matter [30]. The combination forms of HCHs and environmental media are different, which may explain the irregularity of HCHs in the environment. In addition, different forms of HCH can be present as a vapor or adsorbed onto small particles. It can remain in the air for long periods of time, and depending on environmental conditions, residues will travel great distances [19].

### 3.3. Potential Ecosystem Risk Assessment

According to the Environmental Quality Standard for Soil (GB 15618 2018), the risk screening value of ΣHCHs and ΣDDTs is 0.10 mg/kg, and the concentration in all soil samples is lower than the standard value, which indicates that the residue of OCPs in Xiamen is very low. DDT will slowly decompose into DDD and DDE after entering the environment, so the ratio of p, p′-DDE + p, p′-DDD to p, p′-DDT is usually used as the residence time of p, p′-DDT in the environment as indicators of time and degree of degradation [28]. There were three soil samples with a ratio < 1, indicating that there may be new DDT or technical pesticides containing this ingredient used for pest control. The ratio of α-/γ-HCH is also used to reflect the source information of OCPs. There were seven soil samples whose ratio was less than 1, and new HCHs were input on the surface, which may also lead to the irregularity of HCH distribution.

## 4. Conclusions

The distribution and pollution status of OCPs in agricultural soil in Xiamen City were studied. Among them, the range of ΣHCHs was from n.d. to 0.78 ng/g, the average value was 0.076 ng/g, and the detection rate was 28.75%; the range of ΣDDTs was from n.d. to 10.04 ng/g, the average value was 0.232, and the detection rate was 40.83%; The range of ΣOOCPs was from n.d.–3.74 ng/g, the average value was 0.144 ng/g, and the detection rate was 35.71%; the average value of ΣOCPs was 0.149 ng/g, and the detection rate was 35.22%. The results showed that OCPs had a certain degree of detection rate in this soil sample, but the concentration of OCPs was lower than the concentration level of China’s soil environmental quality standards, indicating that the use of these chemicals is decreasing. The distribution of OCPs was significantly positively correlated with soil pH, positively correlated with water content and DOC, and negatively correlated with depth and CEC. Source identification analysis indicated that there were still new inputs of DDTs and HCHs in this region. p, p′-DDT and some cyclodiene pesticides may come from the same pollution source, while HCHs have a separate source, different from other OCPs. Many factors affect the transport process of pesticides in soil; therefore, the bioaccumulation of OCPs during transport and its associated risks to ecosystems and human health require further study.

## Figures and Tables

**Table 1:** Concentrations of OCPs in agricultural soils collected from Xiamen.

Compounds	Agricultural Soils (*n* = 30)		
	Range (ng/g)	Mean (ng/g)	Detection Rate (%)
α-HCH	ND–0.77	0.027	16.7
β-HCH	ND–0.35	0.035	25.0
γ-HCH	ND–0.23	0.017	13.3
δ-HCH	ND–0.78	0.223	60.0
**ΣHCHs**	ND–0.78	0.076	28.8
o, p′-DDT	ND–0.40	0.060	35.0
p, p′-DDT	ND–0.21	0.047	40.0
p, p′-DDD	ND–10.04	0.761	58.3
p, p′-DDE	ND–0.58	0.061	30.0
**ΣDDTs**	ND–10.04	0.232	40.8
Hexachlorobenzene	ND		
Heptachlor	ND–0.86	0.191	66.7
Heptachlor epoxide	ND–0.13	0.003	11.7
Aldrin	ND–0.93	0.157	46.7
Dieldrin	ND–3.74	0.405	48.3
Endrin	ND–1.09	0.195	46.7
Mirex	ND–0.94	0.057	30.0
**ΣOOCPs**	ND–3.74	0.144	35.7
**ΣOCPs**	ND–10.04	0.149	35.2

Mean is the arithmetic mean; ND = Not detected.

**Table 2:** Descriptive statistics of soil properties.

Index	Observed Range	Arithmetric Mean	Standard Deviation
Moisture (%)	10.54–20.00	15.520	3.078
Dissolved organic carbon (mg/g)	7.25–15.19	11.062	1.581
pH	4.34–7.27	5.974	0.848
Cation exchange capacity (cmol/kg)	16.87–39.21	24.107	5.587

## Data Availability

The data used to support the findings of this study are available from the corresponding author upon request.

## Supplementary Materials

The following supporting information can be downloaded at: https://www.mdpi.com/article/10.3390/ijerph20031916/s1, Figure S1: Linear regression studentized residual and Cook distance Analysis of OCPs concentration between soil index; Figure S2: Content vertical distribution of soil indicators at sampling sites; Table S1: Sampling point location; Table S2: Standard curves and detection limits of 15 organochlorine pesticides; Table S3: Linear regression equation analysis of soil index and OCPs.
