# Supplementary material for: Distribution Characteristics and Influencing Factors of Organochlorine Pesticides in Agricultural Soil from Xiamen City
Source: Int J Environ Res Public Health. 2023 Jan 20;20(3):1916. doi: 10.3390/ijerph20031916 (PMC9915912; doi:10.3390/ijerph20031916)
Supplement: Supplementary file 1 [file ijerph-20-01916-s001.zip › ijerph-2157827-supplementary.pdf]

Supplementary Table S1. Sampling point location.

Sampling number	Longitude	Latitude
S1	117°58′12.0″	24°28′21.0″
S2	117°54′57.6″	24°33′28.8″
S3	117°58′33.6″	24°37′12.0″
S4	117°58′37.2″	24°37′8.4″
S5	118°3′32.4″	24°43′58.8″
S6	118°1′1.2″	24°51′43.2″
S7	118°11′2.4″	24°43′40.8″
S8	118°12′3.6″	24°35′31.2″
S9	118°13′1.2″	24°33′57.6″
S10	118°13′12.0″	24°33′7.2″

Supplementary Table S2. Standard curves and detection limits of 15 organochlorine pesticides.

**Compounds**	**Linear Regression Equation**	**R^2**
α-HCH	$y=915.84x-7513.2$	0.9629
β-HCH	$y=684.25x-1425.7$	0.9863
γ-HCH	$y=563.47x-1300.4$	0.9915
δ-HCH	$y=1054x-7027.2$	0.9534
p,p′-DDT	$y=255.81x-1222.9$	0.9787
o,p′-DDT	$y=275.27x+24.647$	0.9899
p,p′-DDD	$y=139.29x+206.06$	0.9883
p,p′-DDE	$y=970.92x+1941.1$	0.9918
Hexachlorobenzene	$y=238.82x+3660.7$	0.9379
Heptachlor	$y=224.17x-919.71$	0.9764
Heptachlor epoxide	$y=857.45x+3383.3$	0.9909
Aldrin	$y=1167.3x+3419$	0.9956
Dieldrin	$y=2.190\times10^{3}x-0.657\times10^{3}$	0.9999
Endrin	$y=0.467\times10^{3}x-0.578\times10^{3}$	0.9995
Mirex	$y=392.98x-130.75$	0.995

Supplementary Note:

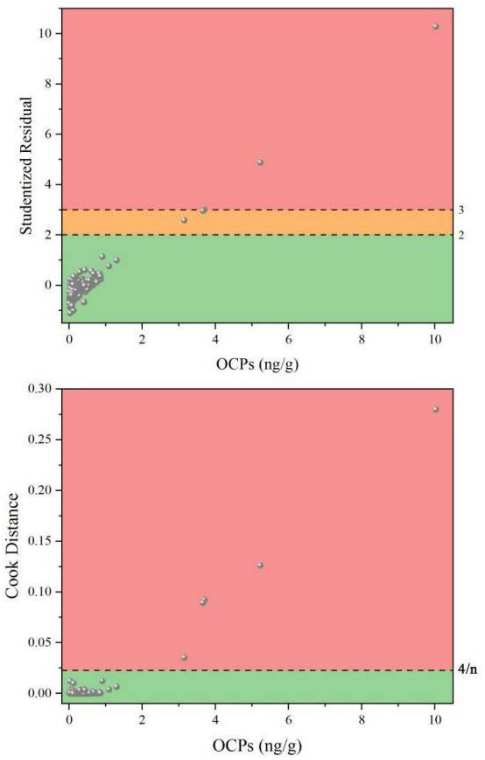

Supplementary Figure S1. Linear regression studentized residual and Cook distance Analysis of OCPs concentration between soil index.

The linearized residuals and Cook distances of the linear regression between the actual measured values and water content, pH, DOC and cation exchange capacity are given in Supplementary Figure S1. The least square method is used for linear regression fitting, which ensures that the sum of squares of the longitudinal distance between each measured point and the straight line is the minimum. If there are outliers or outliers, the regression line has to shift strongly to the direction of the point. Obviously, this may lead to wrong analysis conclusions. The studentized residual is usually an indicator used when testing outliers to reduce the impact of impact points on the residual after model fitting. Generally, "absolute value of studentized residual > 3" is used as the judgment of outliers, and "absolute value > 2" is also used as the standard for statistics of results with small sample number. Similarly, Cook's distance is also a method commonly used in statistical analysis to diagnose whether there are abnormal data in various regression analyses. A large Cook's distance indicates that the coefficients change fundamentally after cases are excluded from the regression statistics and calculations. In this judgment, 4/n is used as the critical value of Cook's distance. In Figure S1, it can be seen that the absolute value of the studentized residual at 5 data points is greater than 2, and 2 of them are significantly far from the data set. In the Cook's distance analysis, there were 181 valid cases, and the calculated critical value was 0.0221. Outside the critical value range, there are still 5 data in the outlier area. Therefore, in the follow-up regression analysis, these 5 data points are counted but not involved in calculation.

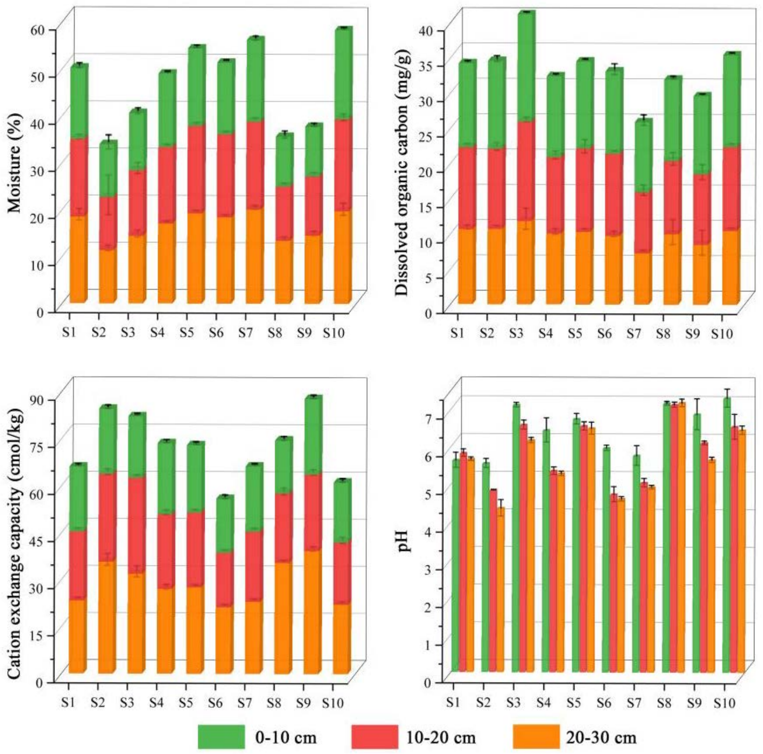

Supplementary Figure S2. Content vertical distribution of soil indicators at sampling sites.

Supplementary Table S3. Linear regression equation analysis of soil index and OCPs.

**Index**	**Category**	**Linear Regression Equation**	**Slop ≠ 0**	**R^2**
Moisture	OCPs	y=0.0129x＋0.043	P < 0.05	0.029
	HCHs	y=－0.007x＋0.320		0.011
	DDTs	y=－0.001x＋0.188		0.001
	OOCPs	y=0.0293x－0.165	P < 0.05	0.110
DOC	OCPs	y=0.0286x－0.081	P < 0.05	0.032
	HCHs	y=0.0039x＋0.175		0.001
	DDTs	y=0.0189x－0.028		0.057
	OOCPs	y=0.0452x－0.230	P < 0.05	0.051
pH	OCPs	y=0.0716x－0.186	P < 0.05	0.067
	HCHs	y=0.0504x－0.071		0.048
	DDTs	y=0.0644x－0.208	P < 0.05	0.132
	OOCPs	y=0.0913x－0.263	P < 0.05	0.077
CEC	OCPs	y=－0.009x＋0.443	P < 0.05	0.036
	HCHs	y=－0.001x＋0.223		0.001
	DDTs	y=－0.002x＋0.238		0.007
	OOCPs	y=－0.018x＋0.069	P < 0.05	0.097
